# Neutrophil to lymphocyte ratio (NLR) in COVID-19: A cheap prognostic marker in a resource constraint setting

**DOI:** 10.12669/pjms.37.5.4194

**Published:** 2021

**Authors:** Kaleem Ullah Toori, Muhammad Arsalan Qureshi, Asma Chaudhry, Muhammad Farhan Safdar

**Affiliations:** 1 Dr. Kaleem Ullah Toori, FRCP (Glasgow). Department of Medicine, KRL Hospital, Islamabad, Pakistan; 2 Dr. Muhammad Arsalan Qureshi (MBBS). Department of Medicine, KRL Hospital, Islamabad, Pakistan; 3 Dr. Asma Chaudhry, MRCP (UK), FCPS General Medicine (Pakistan) Department of Medicine and Endocrinology, Southend University Hospital, Southend-on-Sea, United Kingdom; 4 Muhammad Farhan Safdar, MS. Department of Medicine, KRL Hospital, Islamabad, Pakistan

**Keywords:** COVID-19 RT-PCR, Neutrophil to lymphocyte ratio (NLR), Disease severity (asymptomatic, mild, moderate and severe), Mortality

## Abstract

**Objectives:**

To identify association of neutrophil to lymphocyte ratio with disease severity and mortality.

**Methods:**

Total 720 Corona Virus RT-PCR positive patients were included in this cross-sectional study. Patients were admitted to KRL Hospital Islamabad from April 2020 to August 2020. Neutrophil to lymphocyte ratio (NLR) was recorded on admission and then serially. NLR cut-off was 3.0. WHO categories for disease severity (asymptomatic, mild, moderate and severe) were used. Demographic profile, symptoms and co-morbidities were recorded.

**Results:**

The mean age of patients was 40 ± 12.4 years with 96% being males. Majority patients (76.5%) were asymptomatic. Amongst symptoms, fever was the most common symptom. Diabetes mellitus was most common recorded co-morbidity. The mean NLR 2.5 ± 2.78. Significant association was found between NLR and disease severity as well as mortality. Difference in mean NLR amongst disease severity categories was also significant

**Conclusion:**

Results are compatible with worldwide studies and NLR is a cheap and easily available marker of disease severity and mortality.

## INTRODUCTION

Coronavirus Disease 2019 (COVID-19) is a rapidly spreading disease; first cases of Corona virus emerged in China in 2019, following which it was declared a global pandemic in March 2020 by World Health Organization (WHO).^1^ COVID-19 presents with a wide range of symptoms. The spectrum of disease is variable, with majority cases being mild and self-limiting. However, the disease can be fatal with development of severe pneumonia progressing to acute respiratory distress syndrome (ARDS) and multi-organ failure.^2^

Inflammation plays a major role in development and progression of COVID-19. People infected with COVID-19 are known to have an immune system that is dysregulated and can cause abnormal immune response.^3^ For patients who become septic, timely identification and intervention is necessary to reduce mortality and hospital stay. Circulatory biomarkers which depict inflammation can be used to assess the disease severity and a possible predictor of progression of disease. One such biomarker is Neutrophil to Lymphocyte Ratio (NLR) which can be easily obtained from a simple blood test i.e. Complete Blood Count (CBC) with differential count by dividing the absolute neutrophil count and absolute lymphocyte count. NLR has historically been used as a predictor of morbidity and mortality in patients with cancer,^4^ cardiac disease^5^ and sepsis^6^ amongst other conditions. NLR is a cheap and a readily available indicator of inflammation in patients suffering from COVID-19 and can predict prognosis of patients who are in sepsis.

Studies have been published internationally regarding the use of NLR as a short term prognostic marker in patients with COVID-19 As this is an emerging pandemic, research is rapidly ongoing in order to try to establish how the disease morbidity and mortality can be reduced. We have come across few Pakistani studies^7^,^8^ regarding the use of NLR as a marker of disease severity and prognosis as well. However, as COVID-19 is a huge pandemic necessitating maximal research, we decided to conduct a study in our hospital which caters patients from major cities of Islamabad and Rawalpindi which to our knowledge had not been conducted yet. The purpose of this study was to see if the NLR can be used as a prognostic marker of disease severity and mortality in COVID-19 patients in Pakistani population in a tertiary care hospital.

## METHODS

We designed this as hospital-based, descriptive cross-sectional study. The study took place at KRL Hospital Islamabad. A consecutive series of patients from 1^st^ April 2020 to 31^st^ August 2020 were studied. Non probability, convenience sampling was done. The sample size calculation was not applicable since there is no prevalence data of the disease as the pandemic is still emerging.^9^ The purpose of the study was explained to the patients or their next of kin if the patient was incapacitated and informed consent was obtained. The research was started after being approved by the Hospital’s ethical and research committee (Ref ERC: KRL-HI-ERC/Dec 27, 2020).

All patients admitted with confirmed COVID-19 positive test with at least one nasopharyngeal swab positive for Reverse transcription polymerase chain reaction (RT-PCR) were included in the study. Demographic profile, epidemiological characteristics including possible exposure history, signs and symptoms, comorbidities, drug histories were obtained and recorded. Laboratory investigations and radiological imaging were done as per hospital’s protocol. All X-rays and CT scans were reported by a classified radiologist. NLR was calculated as a simple ratio of absolute neutrophil count and absolute lymphocyte count. NLR cut-off used was 3.^8^ NLR was recorded on admission and then serially.

Case severity definitions were as per the interim guidance of WHO.^10^ Asymptomatic (patients are RT-PCR positive but do not show symptoms), mild (patients are RT-PCR positive but no hypoxia), moderate (RT-PCR positive patients who show signs of pneumonia and but no signs of severe hypoxia with Spo2 > 90%), severe (signs of severe pneumonia evident with respiratory rate more than 30 breaths/minute or Spo2 < 90% and critical (with Acute Respiratory Distress Syndrome [ARDS] or septic shock. We included both severe and critical patients under category of severe.

Statistical analysis was performed using IBM SPSS 25. Continuous variables like age and NLR were reported as mean values with standard deviation. Frequency and percentages were calculated for categorical variables including disease severity categories, symptoms and comorbidities. Pearson’s Chi square test was used to calculate the statistical significance of categorical variables. Confidence interval of 95% was presumed. Tukey’s range test and ANOVA were used to check inter categorical differences in mean values. F statistic was calculated to detect between group variance of mean NLR amongst patient outcome groups i.e. those who recovered or those who died. A p value of < 0.05 was taken as statistically significant.

## RESULTS

A total of 720 patients were admitted during study duration. The mean age was 40 ± 12.4 years (Range- 19 to 87 years). Majority were males i.e. 96% with only 4% being female. Out of the total patients admitted, 76.5% (n=551) were asymptomatic while 23.5% (n=169) were symptomatic. Among the symptomatic, the most frequent symptoms were fever 88.2% (n=149), cough 51.5% (n=87), dyspnoea 36.7% (n=62), myalgia 30.8% (n=52), sore threat 16% (n=27), diarrhoea and vomiting 7.1% (n=12) headache 3% (n=5), rhinorrhea 3% (n=5) and hemoptysis 0.6% (n=1), in order of decreasing frequency.

Out of total patients, 84% (n=606) had no pre-morbidity while 16% (n=114) had underlying chronic medical conditions. The reported premorbid conditions were diabetes mellitus in 8.8% (n=63), hypertension in 7.8% (n=56), ischemic heart disease in 3.1% (n=22), chronic respiratory disease in 2.5% (n=18), chronic kidney disease in 1.7% (n=12), chronic liver disease in 0.6% (n=4), chronic neuro-psychiatric condition in 0.2% (n=2) and malignancy in 0.1% (n=1).

Regarding disease severity, 76.5% (n=551) were asymptomatic, 13.3% (n=96) had mild disease, 4.6% (n=33) had moderate disease and 5.6% (n=40) had severe disease. Amongst the total patients, 97.8% (N=704) recovered while 2.2% (n=16) died.

The mean NLR of 720 patients was 2.5 ± 2.78 (Range- 0.14 to 31.33). The descriptive statistics of NLR across all four categories of disease severity are reported in Table-I. In summary, mean NLR shows a rising trend from 1.92 in asymptomatic patients to 2.08 in mild, 4.79 in moderate and 9.9 in severe patients, signifying a positive association between NLR and disease severity in our sample.

**Table-I:** Variability of NLR across all categories of disease severity – ANOVA.

		Disease Severity			

		Asymptomatic	Mild	Moderate	Severe
NLR	Mean (95% Confidence Interval)	1.92 (1.8, 2)	2.08 (1.83, 2.32)	4.79 (3.79, 5.8)	9.9 (7.79, 12)
	Minimum	0.14	0.65	1.53	2.46
	Maximum	15	7.36	14.83	31.33
	Std. Dev	1.3	1.19	2.83	6.58
	n	551	96	33	40
Pearson χ^2^ value=1656.3 d.o.f =1497 p-value=0.02					

d.o.f: degree of freedom.

To further explore the difference in mean NLR across various categories of disease severity, Tukey’s range test was applied. The results are reported in Table-II. The difference in mean NLR is statistically significant in all the categories except asymptomatic and mild.

**Table-II:** Difference in mean NLR amongst disease severity categories- Tukey’s range test.

	Mean Difference	Standard Error	p-value	Lower bound	Upper bound
Asymptomatic- Mild	-.16	.227	.894	-.746	.424
Asymptomatic-Moderate	-2.87*	.368	.000	-3.827	-1.930
Asymptomatic-Severe	-7.98*	.336	.000	-8.850	-7.117
Mild-Moderate	-2.71*	.414	.000	-3.785	-1.649
Mild-Severe	-7.82*	.386	.000	-8.819	-6.827
Moderate-Severe	-5.10*	.483	.000	-6.349	-3.860

*Note:*

* shows mean difference is significant at 0.05 level.

The mean NLR of patients who recovered from COVID-19 compared with those who died are presented in Table-III. To statistically test the difference in mean NLR between both groups, ANOVA was conducted. It was quite evident that patients who died had a significantly elevated NLR compared to those who recovered.

**Table-III:** Association between NLR and mortality- ANOVA.

	Mean NLR	Minimum	Maximum	F-Statistic, p-value
Recovered	2.34 ± 2.25	0.14	19	164.27, 0.00
Died	10.5 ± 8.11	3.17	31.33	

## DISCUSSION

COVID-19 is a highly infectious disease which is an ongoing immense threat to global public health. It has been rapidly spreading throughout world with a second peak now evident in many countries.^11^ Even though majority patients have self-limiting and mild illness, patients who develop severe or critical cases have a grave prognosis. It had been observed very early after the beginning of COVID-19 pandemic that the neutrophil-to-lymphocyte ratio (NLR) is much higher in severe or critically ill patients as compared to those with milder disease. NLR has been shown to be a reliable indicator to determine disease severity in COVID-19.^11^,^12^ Many mechanisms have been postulated regarding the response of neutrophils and lymphocytes to corona virus infection. Neutrophils activate the immune system and release reactive oxygen species that can induce cell DNA damage and release the virus from the cells which is then targeted by antibodies. In addition, neutrophils trigger the production of various cytokines and effector molecules. On the other hand, although the viral infection itself triggers lymphocyte response predominantly, the systemic inflammation especially high Interleukin 6 paradoxically decreases the lymphocyte count and resultant cellular immunity. Both these factors result in elevated NLR.^12^ Hence a higher NLR predicts the severity of inflammation.

The mean age of patients developing COVID is variable. The mean age in our population was 40 years which is lower as compared to other Pakistani studies.^8^,^13^ Our patients were predominantly males which is keeping with other studies as COVID-19 is known to have a predilection for male gender.^8^ The patients in our study were mostly asymptomatic which is similar to data acquired by WHO suggesting up to eighty percent patients maybe asymptomatic.^14^ Amongst the symptomatic individuals, the most common clinical symptoms were fever, respiratory symptoms, and myalgias which are comparable to those described by the WHO interim guidance.

Our study has shown that mean NLR value significantly increases as disease severity progresses with lowest NLR recorded in asymptomatic and mild disease. This is in line with a Cochrane Meta-analysis Review of twenty Chinese studies which established that NLR is an independent prognostic marker to differentiate severe vs non-severe COVID-19 disease.^15^ Our results are also consistent with an Italian study with 74 patients in whom severe cases had an NLR of 5.6 vs 3.0 in non-severe cases.^2^ Another meta-analysis also endorsed our results establishing that NLR levels are directly proportional to disease severity in patients.^16^ Pakistani studies conducted in different cities by Pervaiz et al^7^ and Asghar et al^8^ have also found similar findings. According to a Chinese study, it was suggested that NLR is superior in early prediction of severe and critical illness when compared to widely used scoring severity criteria accepted for pneumonia namely the CURB-65 Score (which evaluate 30-day mortality of patients with community-acquired pneumonia) and MuLBSTA Score (which gives early warning about the mortality of patients with viral pneumonia.^15^ We also found NLR on admission to be a reliable predictor of disease severity.

We reviewed the difference in mean NLR between disease categories as well and found that the highest difference in mean NLR is between the asymptomatic and severe patients. This was expected and in keeping with our findings of strong association between mean NLR and severe disease.

We established a link between high NLR and mortality as well in our study. The sixteen patients that succumbed to the disease in our center had a mean NLR ratio of 10.5 as compared to 2.34 in those who recovered which was significantly high. This association between high NLR and mortality has been observed both nationally^7^,^8^ and internationally.^11^,^12^ Yan X and Colleagues showed that high NLR is an independent risk factor causing in hospital morality in COVID-19 patients.^17^

Our study results are consistent with the above mentioned studies supporting the theory that NLR is a cheap, robust and easily available predictor of COVID-19 disease morbidity and mortality. It’s a simple marker giving objective evidence of patients at risk of severe disease and increased mortality.

### Limitations of the Study

Firstly, it was conducted at a single center. Secondly predominantly male patients as being employees of the organization all males were required to be admitted if they tested positive for RT-PCR. On the other hand, female patients with mild disease opted to isolate at home and only admitted if they had moderate to severe disease requiring hospital management.

## CONCLUSION

In our study we highlight the importance of NLR in COVID-19 patients in predicting disease severity and mortality. In a developing country like ours where there are resource limited settings, NLR can be used as an effective marker to predict and stratify COVID-19 patients as per severity and effectively predict the outcome as well, which in turn would lead to efficient resource utilization.

### Authors Contribution:

**KT:** Conceived, designed and did statistical analysis along with review of manuscript. He is also Responsible for accuracy of data collection and integrity of work.

**MAQ:** Contributed to data collection and manuscript writing.

**AC:** Did manuscript writing with final review of manuscript.

**MFS:** Did data collection and entry.
